# Environmental Impact of Pentaspline Pulsed Field Ablation–Global Warming or Arctic Front?

**DOI:** 10.36469/001c.157672

**Published:** 2026-02-24

**Authors:** Peter Calvert, Vishal Luther, Dhiraj Gupta

**Affiliations:** 1 Department of Cardiology Liverpool Heart & Chest Hospital, Liverpool, UK; 2 Liverpool Centre for Cardiovascular Science at University of Liverpool, John Moores University, Liverpool, UK

We read with interest the study by Chun et al^1^ regarding the environmental impact of pulsed field ablation (PFA) compared with cryoballoon (CB) ablation for atrial fibrillation (AF). The authors note the importance of minimizing the carbon footprint of interventional procedures –a concept with which we thoroughly agree. The environmental impact of anesthetic gases is well described.^2^ The authors reported that PFA was more environmentally friendly, largely driven by differences in anesthetic gas use, specifically sevoflurane.

We must, however, question the parameters used for input into the model, particularly with reference to the United Kingdom National Health Service (UK NHS). According to Table 1 of the manuscript, the authors’ model applied sevoflurane use in over 66% of CB cases, but fewer than 20% of PFA cases. The reference given in support of this is an abstract from the PERFECT-PAF randomized controlled trial; however, this abstract gives no details on the type of anesthesia used.

Moreover, in the UK it is standard practice for pentaspline PFA cases to be performed under general anesthetic using sevoflurane, while CB cases are typically performed under mild conscious sedation using midazolam and fentanyl without any sevoflurane. In our own published data from an NHS cardiac tertiary center, general anesthesia was utilized in 100% of 208 PFA ablations, but just 10% of 325 CB procedures.^3^ These data were from our early experience of PFA and given the benefits in terms of procedure time, anecdotally, very few operators would now perform CB under full general anesthetic when PFA could be used instead. Sensitivity analyses by Chun et al did not test for such a scenario; while they ran a simulation for total intravenous anesthetic, this was assumed to be the case in both arms, whereas a simulation reflective of real-world UK practice would need to assume inhaled anesthetic in 100% of PFA cases but 0% of CB cases. This would almost certainly make CB more environmentally friendly if anesthetic gas is the primary driver. This highlights the need for further research into PFA systems which may be better tolerated under conscious sedation,^4^ or alternative anesthetic strategies.

Overall, while we are enthusiastic about PFA in terms of workflow, efficiency, and clinical outcomes, we do not believe the statistical modeling in the current study is accurately reflective of UK NHS practice. Efforts to reduce environmental impact of PFA systems would be welcomed.

## Disclosures

P.C. has no conflicts to declare. V.L. received support from UK National Institute for Health Research scholarship award, was speaker for Boston Scientific and J&J MedTech, and received research grants from J&J MedTech. D.G. received institutional research grants from J&J MedTech, Boston Scientific, and Medtronic, and speaker fees from Boston Scientific.
